# Anthropometric Data

**Published:** 1916-04

**Authors:** 


**Anthropometric Data.** E. A. Hooton, Cambridge, Mass., Boston M. & S. Jour., Jan. 27, denying the existence of carnivorous and herbivorous types of man, gives the following tables:

(Males). After Martin.

| | Stature. | Weight. | Index of Bodily Fullness |
|---|---|---|---|
| Europe | | | |
| Norwegians | 170.0 | 66.0 | 1.29 |
| Polish Jews | 161.0 | 55.0 | 1.31 |
| Swiss (Schaffhausen) | 169.4 | 65.8 | 1.35 |
| South Russian Jews | 165.1 | 61.3 | 1.42 |
| Asia | | | |
| Javanese | 163.5 | 50.2 | 1.15 |
| Japanese | 162.0 | 52.0 | 1.22 |
| Sudanese | 159.4 | 51.5 | 1.27 |
| Koreans | 163.1 | 56.4 | 1.37 |
| North Chinese | 167.6 | 64.4 | 1.37 |
| Africa | | | |
| Bushmen | 155.4 | 40.4 | 1.07 |
| Baluba | 169.0 | 53.3 | 1.10 |

| | 25 Pre-sacral Vertebrae. | 23 Pre-sacral Vertebrae | |
|---|---|---|---|
| 908 Europeans | 4.3% | 1.2% | (Bardeen) |
| 524 Europeans | 6.2% | 2.6% | (Fischel |
| 640 Europeans | 3.9% | 4.7% | (Rabl) |
| 358 Europeans | 5.0% | 3.0% | (Keith) |
| | 13 Ribs. | 11 Ribs. | |
| 524 Europeans | 6.6% | 0.5% | (Fischel) |
| 680 Europeans | 6.2% | 0.3% | (Rabl |
| 908 Europeans | 0.7% | 0.9% | (Bardeen) |

| | Average Height (Males) |
|---|---|
| Africa | |
| Dinka, Nuer | 180.0 cm. |
| Sara | 181.7 cm. |
| Shilluk | 177.7 cm. |
| Baghirmi, Wadi, Fur | 178.0 cm. |
| Europe. | |
| Scotchmen | 174.6 cm. |
| Englishmen | 172.8 cm. |
| Swedes | 170.9 cm. |
| Italians | 163.0 cm. |
| Spaniards | 162.0 cm. |

